# Retrospective study examining complications and iatrogenic pseudopregnancy in bitches neutered in different stages of the oestrous cycle: identification of an ‘early neutering window’ in bitches

**DOI:** 10.3389/fvets.2026.1774042

**Published:** 2026-02-03

**Authors:** Rachel Moxon, Gary C. W. England

**Affiliations:** 1 Guide Dogs National Centre, Warwickshire, United Kingdom; 2 School of Veterinary Medicine and Science, University of Nottingham, Leicestershire, United Kingdom

**Keywords:** bitch, dog, intraoperative bleeding, neuter, neuter timing, pseudopregnancy

## Abstract

**Introduction:**

Neutering in the anoestrus period is most commonly recommended for bitches due to reduced risk of intraoperative bleeding and iatrogenic pseudopregnancy. However, there is a possible second period for neutering, commencing after oestrus until the time when prolactin concentrations increase.

**Methods:**

This retrospective study compared reports of intraoperative bleeding (categorised in two ways: excluding and including bleeds from the skin and subcutaneous tissue/fat) and cases of pseudopregnancy between bitches neutered during an ‘early’ period (EP bitches, neutered from the end of oestrus until day 43 after ovulation, *n* = 319), during a ‘conventional’ period (CP bitches, neutered 90 or more days from estimated ovulation date, *n* = 1,993) and bitches neutered in an ‘at greatest risk’ period (GRP bitches, neutered between 44 and 89 days from estimated ovulation date, *n* = 231). Data for pseudopregnancy post-neutering were gathered using two methods (1) clinical indicators from electronic health records and (2) routinely collected behavioural data for behaviours that have been associated with pseudopregnancy.

**Results:**

There was no association between neuter period and the presence of an intraoperative bleed, either when excluding or including bleeding from the skin and subcutaneous tissue/fat (3.8–4.4% EP, 5.8–8.8% CP, and 7.4–11.3% GRP bitches). Only six cases of pseudopregnancy after neutering were identified in clinical records: two EP bitches (0.6%), one CP bitch (0.05%) and three GRP bitches (1.3%); associations were not evaluated statistically due to the small number of cases. Examinations of behavioural data found no association between neuter period and trait scores for ‘General Anxiety’, ‘Body Sensitivity’ and ‘Separation-related behaviours’ from dog behaviour questionnaires. However, EP (4.1%) and CP bitches (11.7%) had reduced odds of having a behaviour incident and/or development plan related to fear or anxiety and/or aggression post-neutering compared to GRP bitches (20.8%).

**Conclusion:**

The results suggest that neutering bitches during the ‘early’ period may be a suitable alternative to the ‘conventional’ period. Additionally, the results support the generally well-accepted advice to avoid neutering bitches during the period of greatest risk.

## 1 Introduction

It is important to consider the timing of surgical neutering for bitches in relation to the previous oestrus. During proestrus and oestrus, under the influence of oestrogen, the bitch’s reproductive tract is vascular (1, 2), and therefore there may be increased risk of intraoperative haemorrhage for neutering procedures undertaken during this period (3). In the second part of the luteal phase, plasma prolactin concentrations increase, commencing approximately 25 days post-ovulation in pregnant bitches and 43 days post-ovulation in nonpregnant bitches (4), although the increase is much greater for pregnant bitches. Prolactin remains increased until the end of the luteal phase at around 70 days after ovulation. Prolactin is luteotrophic and increases in response to decreasing concentrations of progesterone (5–8). Removal of the ovaries during the luteal phase, when prolactin concentrations are elevated, causes a rapid decrease in progesterone, which can cause an increase in prolactin (9), and risks inducing an iatrogenic pseudopregnancy. Prolactin may still be elevated after the end of the non-pregnancy luteal phase, up to day 90 or so after ovulation, and these bitches may have signs of spontaneous pseudopregnancy. Removal of the ovaries at this time (day 70–90) does not cause iatrogenic pseudopregnancy (because pseudopregnancy already exists) and does not make the pseudopregnancy worse (there is no decrease in progesterone and so no further stimulation of prolactin).

The mechanisms for reduction of prolactin to basal levels after the end of the luteal phase are unknown but may involve ovarian factors. It is plausible then that removal of the ovaries, which causes iatrogenic pseudopregnancy, or removal of the ovaries at the time of spontaneous pseudopregnancy, may result in a persistence of elevated prolactin, which might allow pseudopregnancy to persist. For these reasons it is common practice to delay removal of the ovaries until bitches are clearly in the anoestrus phase of the cycle (usually spanning the period from at least 90 days after ovulation until the beginning of the next proestrus) (2, 10).

Based upon the endocrinological features of the oestrous cycle of the bitch, we hypothesise that there is a further period of opportunity for surgical neutering for the non-pregnant bitch, commencing after oestrus (when the tract is no longer vascular) and extending until the time when prolactin concentrations increase (and there is a risk of inducing iatrogenic pseudopregnancy). Two previous studies including small numbers of bitches (between six and 18) have been undertaken to investigate the effects of surgical neutering in mid-dioestrus on prolactin concentrations and the occurrence of pseudopregnancy. In bitches with no history of pseudopregnancy, prolactin concentrations were higher after neutering, but no overt signs of pseudopregnancy were observed (9, 11). Our hypothesis for this early neutering window is based upon targeting neutering in the early to mid-dioestrus period.

The aim of this retrospective study was to compare the complications of intraoperative bleeding and cases of pseudopregnancy post-neutering for bitches neutered during this ‘early’ period (from the end of oestrus until day 43 after ovulation; early to mid-dioestrus) with bitches neutered in the ‘conventional’ period (anoestrus). The specific objectives were to determine the presence of an intraoperative haemorrhage and to determine the presence of clinical pseudopregnancy or observed behaviours that may be indicative of pseudopregnancy following neutering surgery for these two groups. In addition, some bitches were identified that were neutered in the period of ‘greatest risk’ (mid to late dioestrus), and their data were also included for comparison.

## 2 Materials and methods

### 2.1 Study design, setting and animals

A retrospective study was undertaken using data extracted from electronic health records for bitches in a large assistance dog programme. Data were collated for non-pregnant bitches that were neutered between 01/01/2018 and 18/10/2024. Bitches in the assistance dog programme are routinely neutered during puppy raising, before entering formal training at 12 to 14 months of age, or, for bitches in the breeding programme, on leaving the programme. The organisation’s electronic database is used to record data on all health events for all dogs. This includes veterinary notes relating to neutering surgeries (including complications such as intraoperative bleeds, the reporting of which is reliant on the operating surgeon submitting details in their clinical notes), season dates and disease diagnoses, including pseudopregnancy. Behavioural data are also recorded for dogs as they progress though the training programme, including on behavioural questionnaires completed at five, eight and 12 months of age, and at four and 12 weeks into training or being accepted on the breeding programme. Additionally, behaviour incident reports and dog development plans are completed for dogs on an *ad hoc* basis when abnormal behaviours are observed.

### 2.2 Variables and data sources

#### 2.2.1 Neutering surgery health notes

Neutering surgery health records for 3,182 individual bitches were extracted by using the reporting function in the electronic database to identify records containing the health codes: ‘Routine spay’, ‘Ovariectomy [midline]’, ‘Ovariectomy [via laparoscopic technique]’, and ‘Ovariohysterectomy [via laparoscopic technique]’. Entries relating to male dogs (*n* = 2), emergency/medically abnormal surgeries (*n* = 5) and surgeries for ovarian remnants (including the original surgery resulting in the ovarian remnant, *n* = 6) were removed (see Supplementary material). The bitch’s body condition score (within 60 days of neutering surgery date) and the veterinary practice performing the surgery were also extracted.

#### 2.2.2 Length of time from previous oestrus to neutering surgery

The database’s reporting function was used to identify records containing the health codes ‘Season start’ and ‘Season end’. Associated season start and end dates were extracted for all bitches with neutering surgery health records, and duplicates were removed to retain the most recent season dates. For bitches in the dataset with missing season date information, the dog’s electronic records were manually searched and season dates were extracted when identified. Any bitches that were indicated in the health records to have been neutered without an observed season were identified. Any bitches with a season start but no season end date recorded were assumed to have a season length of 21 days and this was used to calculate season end date (*n* = 564). Health records for any bitches that appeared to have long intervals (>270 days) between the previous season and neutering surgery were examined to confirm the absence of an intervening season. Season dates were recorded as ‘Unknown’ for any bitch where season start and end dates were not identified. Day of ovulation was estimated as season end date minus 7 days (12). The length of time between the neutering surgery date and estimated ovulation date in days was calculated.

#### 2.2.3 Intraoperative bleeding

All extracted health record notes relating to the neutering surgery were manually examined to identify those that mentioned a bleed or oozing during surgery or in the immediate postoperative period. The site of the bleed was noted where stated. Bleeds were categorised in two ways: including bleeds from the skin and subcutaneous tissue/fat and excluding bleeds from the skin and subcutaneous tissue/fat.

#### 2.2.4 Pseudopregnancy

The database’s reporting function was used to identify records containing the health code ‘False pregnancy’ for pseudopregnancies that occurred from 01/01/2008 to 31/12/2024. Bitches that have experienced multiple seasons, particularly breeding bitches, may have experienced multiple pseudopregnancies. For each bitch, whether they had a pseudopregnancy or not was noted, along with the date that the pseudopregnancy was first mentioned, where applicable. The number of days between pseudopregnancy and neutering surgery date was calculated and whether the pseudopregnancy occurred before or after neutering surgery was recorded.

#### 2.2.5 Pseudopregnancy post-neuter

Data for pseudopregnancy post-neutering were gathered using two methods (1) clinical indicators from electronic health records and (2) routinely collected behavioural data.

##### 2.2.5.1 Clinical indicators from health records

Health records were extracted for bitches with health codes for ‘false pregnancy’ reported at any time after neutering surgery. In addition, for all bitches with a pseudopregnancy in the 6 months before neutering surgery, health records were examined to determine whether any clinical signs of pseudopregnancy were observed in the first month following neutering (signs searched for included mammary gland enlargement with or without the production of milk, inappetence, lethargy, periodic vomiting, [excluding single episodes in the days follow general anaesthetic], nesting behaviours and fluid retention).

##### 2.2.5.2 Behaviours that may indicate pseudopregnancy

Behavioural data are routinely collected for dogs using behaviour questionnaires, which collect data on nine behavioural traits (13). Three of these traits, ‘General Anxiety’, ‘Body Sensitivity’ and ‘Separation-related behaviours’ were considered those that may be observed in bitches experiencing pseudopregnancy (10, 14, 15). Trait scores for these three traits were extracted where available for all bitches in the study from puppy and dog behaviour questionnaires (PBQ/DBQ) completed at 12 months of age, and at four and 12 weeks into training. Only questionnaires completed between 2 weeks and 6 months after neutering were included.

In addition, data relating to behaviours associated with these three traits were extracted from ‘Behaviour Incident reports’ and from ‘Dog Development Plans’; these are tools that are used within the organisation to record one off behavioural observations or plans for addressing certain behavioural concerns. Any behaviour incident reports and/or development plans that were identified for bitches in the 6 months following neutering were reviewed for the presence of behaviours associated with fear or anxiety, and/or aggressive behaviours.

### 2.3 Bias and study size

Study size was limited to the number of bitches with neutering and season data available for inclusion in the study. Due to the retrospective nature of the study design, the number of different veterinary practices that undertook neutering surgeries was large (*n* = 801), with 72.5% of practices undertaking only one or two surgeries each, therefore a grouped veterinary practice variable was included with individual practices that had <30 surgeries in the dataset grouped as ‘Other’. High levels of abdominal fat in overweight bitches and high BCS have been suggested to be associated with intraoperative difficulties during neutering (16, 17), therefore bitch BCS was included in models examining the presence of intraoperative bleeds. Behaviour questionnaire scores have been shown to vary between breeds of dog (18–22); therefore, breed was included in all statistical models.

### 2.4 Quantitative variables and statistical methods

The outcome variables were the presence of an intraoperative bleed, the presence of clinical signs associated with pseudopregnancy and the presence of behaviours that may indicate pseudopregnancy. Bitches with missing data for season dates, and that were neutered without experiencing a season, were excluded from analysis due to the inability to calculate time between season and neutering surgery, which was required to determine neutering period, the primary explanatory variable. Bitches were placed into one of three neutering categories based on the timing of neutering in relation to the previous oestrus: ‘early’ period (EP) bitches (neutered from the end of oestrus until 43 days from the estimated ovulation date; early to mid-dioestrus), ‘conventional’ period (CP) bitches (neutered 90 or more days from the estimated ovulation date; anoestrus), or the ‘at greatest risk’ period (GRP) bitches (neutered 44 to 89 days from the estimated ovulation date; mid to late dioestrus). These categories were based on prolactin concentrations reported for nonpregnant bitches by Onclin and Verstegen (4).

Categorical covariates included in all statistical models were examined using descriptive statistics and any that had fewer than 30 bitches were identified and grouped. Mean ± SEM and 95% CI were reported where appropriate. The area under the ROC curve (AUC) was reported for binary logistic regression models. Data were analysed using XLStat 2021.3.1 and SPSS version 28.

#### 2.4.1 Intraoperative bleeding

Binary logistic regression models using stepwise backward elimination were used to examine the impact of neutering period on the presence of an intraoperative bleed while controlling for potential confounding variables of breed, BCS, veterinary practice (VP) (grouped as VP1 (*n* = 93), VP2 (*n* = 38), VP3 (*n* = 482), VP4 (*n* = 39) and ‘Other VPs’ (*n* = 1,891)) and age at neutering. Two models were run: one including and one excluding bleeds/oozing from the skin and subcutaneous tissue/fat. The response variable was the presence or absence of an intraoperative bleed (1, 0). Breed (grouped, Table 1), BCS (grouped as ideal [BCS 4 or 5], overweight [BCS 6 or more] or ‘unknown’ BCS), veterinary practice (grouped) and age at neutering were included as explanatory variables. Variables with *p* < 0.2 were retained in the models.

**Table 1:** The breed groups for the 2,543 bitches with neutering records included in the data analysis.

Bitch breed	*N*	Percentage
German shepherd dogs and crosses	241	9.5
Golden retriever (GR)	269	10.6
Labrador retriever (LR)	726	28.5
First generation GR x LR or LR x GR	800	31.5
Second generation (or more) GR x LR or LR x GR	462	18.2
Other	45	1.8

#### 2.4.2 Indicators of pseudopregnancy

##### 2.4.2.1 Clinical indicators

The number of bitches that had health codes for ‘false pregnancy’ at any time after neutering surgery was described. Data analysis was not possible due to small numbers of bitches reported with clinical signs; data were described in relation to days between estimated ovulation date and neutering surgery.

##### 2.4.2.2 Behaviours that may indicate pseudopregnancy

Associations between neutering period category and trait scores for ‘General Anxiety’, ‘Body Sensitivity’ and ‘Separation-related behaviours’ from four- and 12-week DBQ were analysed using generalised linear models, one for each trait at each age. Neutering period category and bitch breed group were included as fixed factors in all models. Days of age at questionnaire completion and days between neutering surgery and questionnaire completion were also included as covariates. All models were fitted using IBM SPSS Statistics for Windows, version 28 (IBM Corp., Armonk, N. Y., USA).

Data for behaviour incident reports and dog development plans were reported and compared between EP, CP and GRP bitches. The number of bitches with and without a behaviour incident report and/or dog development plan for fear/anxiety or aggression-related behaviours in the 6 months following neutering were compared using a binary logistic regression model using stepwise backward elimination while controlling for the potential confounding variables of breed and age at neutering. The response variable was the presence or absence of a behaviour incident report and/or dog development plan (1, 0). Breed group and age at neutering were included as explanatory factors.

#### 2.4.3 Indicators of pseudopregnancy post-neuter for bitches that experienced a pseudopregnancy after the oestrus prior to neutering

Clinical signs associated with pseudopregnancy that were identified following neutering surgery for bitches that had experienced a pseudopregnancy after the oestrus immediately prior to neutering were examined and described.

## 3 Results

### 3.1 Study animals

Neutering records were available for a total of 3,169 bitches, which were born between 2010 and 2023. Bitches were either pure or crossbreeds of curly coated retriever, golden retriever (GR), Labrador retriever (LR), German shepherd dog (GSD) and standard poodle. Three hundred and ninety-four (12.4%) of the bitches had pseudopregnancies reported that occurred at some point prior to neutering (379 bitches experienced one pseudopregnancy, nine bitches experienced two and four bitches experienced three pseudopregnancies). Seven bitches (0.2%) had pseudopregnancies reported after neutering (two of these seven bitches had previously experienced a pseudopregnancy; these were not associated with the oestrus immediately prior to neutering).

Two hundred and fifty-five bitches (8.0%) were reported to have an intraoperative bleed when bleeds/oozing from the skin and subcutaneous tissue/fat were included, and 171 (5.4%) were reported to have an intraoperative bleed when bleeds/oozing from the skin and subcutaneous tissue/fat were not included.

Season dates were not identified for 530 of the 3,169 bitches, and 96 bitches were neutered without having an observed season. These 626 bitches were excluded from subsequent analyses (see Supplementary material). The remaining 2,543 bitches were neutered between seven and 440 days (mean = 100.2 ± 0.7 days) from the estimated previous ovulation date. Data from these bitches were used to examine neutering timing in relation to the previous oestrus.

Bitches were grouped into six breed groups (Table 1) and were aged between 0.8 and 8.0 years (mean = 1.8 ± 0.04 years) at the time of neutering. BCS data were available for 1,374 bitches within 60 days of neutering surgeries; 987 bitches had a BCS representing being an ideal weight and 383 had a BCS representing being overweight.

**Figure 1:**
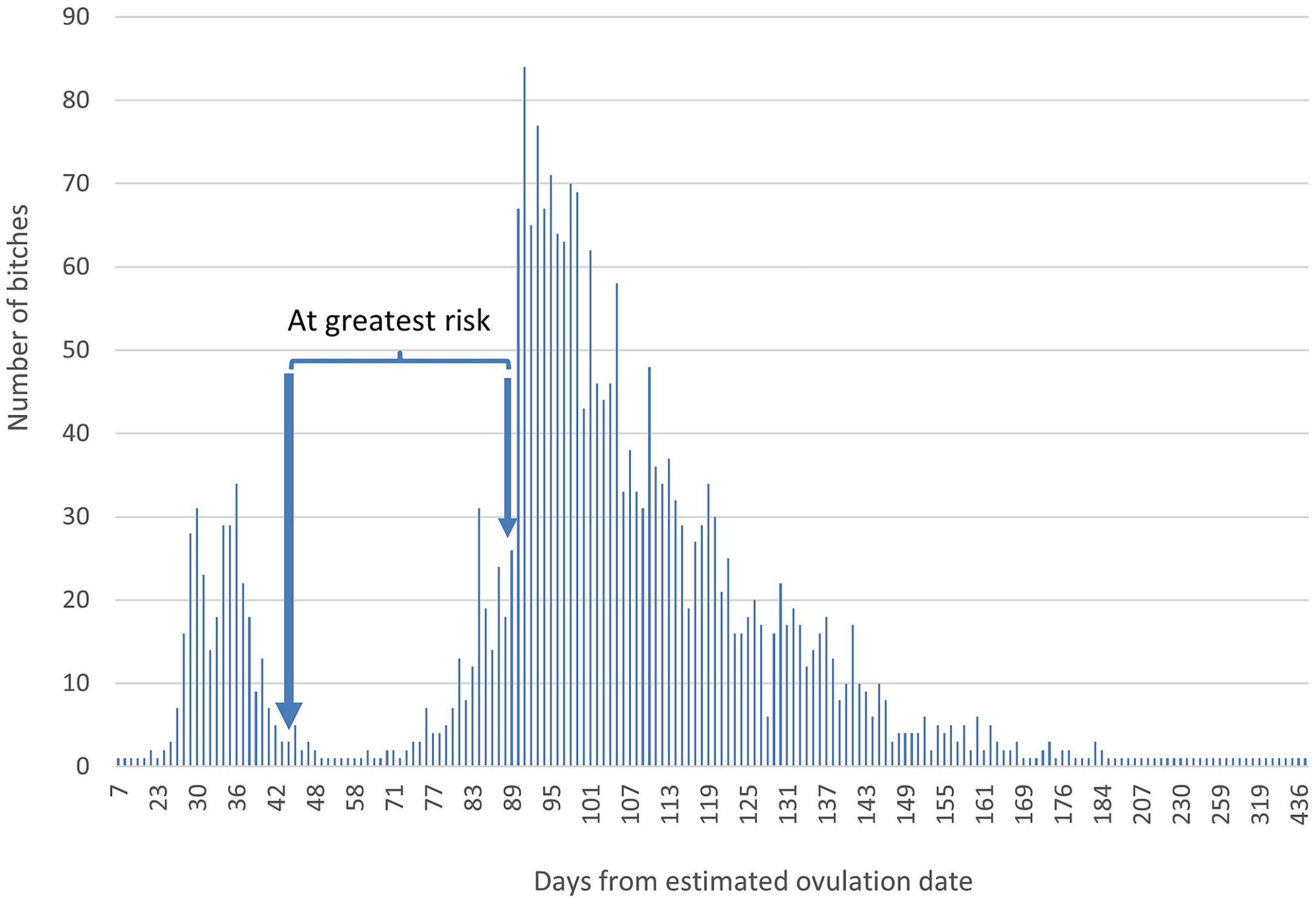
The number of bitches neutered each day from estimated ovulation date. Arrows indicate the start and end of the period where neutering poses the greatest risk to bitch health.

When bitches were categorised based on the timing of neutering surgeries, there were 319 EP bitches, 1,993 CP bitches and 231 GRP bitches (Figure 1). Forty EP bitches (12.5%), 276 CP bitches (13.8%) and 16 GRP bitches (6.9%) had a history of at least one pseudopregnancy prior to neutering.

### 3.2 The presence of an intraoperative bleed

Two hundred and sixteen (8.5%) of the 2,543 bitches had an intraoperative bleed reported when including bleeding or oozing from the skin and subcutaneous tissue/fat, and 144 (5.7%) had an intraoperative bleed reported when bleeds/oozing from the skin and subcutaneous tissue/fat were not included (Table 2).

**Table 2:** Descriptive data for categorical variables included in the regression models for surgeries that had an intraoperative bleed reported when including and excluding bleeding or oozing from the skin and subcutaneous tissue/fat.

Variable	Bitches with an intraoperative bleed reported (*n*, %) including bleeding or oozing from the skin and subcutaneous tissue/fat	Bitches with an intraoperative bleed reported (n, %) excluding bleeding or oozing from the skin and subcutaneous tissue/fat
Neutering period group		
Conventional	176/1993–8.8%	115/1993–5.8%
Early	14/319–4.4%	12/319–3.8%
At greatest risk	26/231–11.3%	17/231–7.4%
Body condition score group		
Ideal	95/987–9.6%	62/987–6.3%
Overweight	26/383–6.8%	19/383–5.0%
Unknown	95/1173–8.1%	63/1173–5.4%
Breed group		
Golden retriever (GR)	17/269–6.3%	13/269–4.8%
German shepherd dogs and crosses	20/241–8.3%	16/241–6.6%
Labrador retriever (LR)	67/726–9.2%	48/726–6.6%
First gen GRxL or LxGR	70/800–8.8%	42/800–5.3%
Second gen GRxL or LxGR	38/462–8.2%	22/462–4.8%
Other	4/45–8.9%	3/45–6.7%
Veterinary practice group		
VP1	2/93–2.2%	2/93–2.2%
VP2	4/38–10.5%	4/38–10.5%
VP3	10/482–2.1%	7/482–1.5%
VP4	2/39–5.1%	2/39–5.1%
Other VPs	198/1891–10.5%	129/1891–6.8%

When the data included bleeding or oozing from the skin and subcutaneous tissue/fat, the regression model was significant (D. F. = 7, Chi-square = 63.427, *p* < 0.001, AUC = 0.640). Neutering period (EP/CP/GRP) and bitch breed group were not retained in the final model. Bitch BCS (D. F. = 2, Chi-square = 8.970, *p* = 0.012) and veterinary practice (D. F. = 4, Chi-square = 13.666, *p* < 0.001) were significant. Bitches that had an unknown BCS (8.1% with bleeds, OR = 0.634, 95% CI 0.467–0.859, *p* = 0.003) had reduced odds of having a bleed reported compared to bitches that had an ideal BCS (9.6% with bleeds). Bitches neutered at VP1 (2.2% with bleeds; OR = 0.161, 95% CI 0.039–0.663, *p* = 0.011) and at VP3 (2.1% with bleeds; OR = 0.307, 95% CI 0.124–0.761, *p* = 0.011) had reduced odds of having a bleed reported than bitches neutered at ‘Other’ VPs (10.5% with bleeds).

When the data excluded bleeding or oozing from the skin and subcutaneous tissue/fat, the regression model was significant, however neutering period was not retained in the final best fit model (D. F. = 12, Chi-square = 44.458, *p* < 0.001, AUC = 0.647). Veterinary practice (D. F. = 4, Chi-square = 7.323, *p* = 0.044) was the only significant explanatory variable. However, *p* > 0.05 was observed for all individual comparisons between VPs. No other variables were significant. The equations for the best fit models are provided in the Supplementary material.

### 3.3 Indicators of pseudopregnancy

#### 3.3.1 Clinical indicators from health records

Six of the 2,543 bitches (0.2%) had health codes for ‘false pregnancy’ after neutering, first recorded between three and 10 days post-surgery. This represents 0.6% of EP bitches, 0.05% of CP bitches and 1.3% of GRP bitches. No cases were associated with a pseudopregnancy following the oestrus immediately prior to neutering, however one EP bitch and one GRP bitch had experienced a pseudopregnancy after a previous oestrus. Days between estimated ovulation date and neutering surgery for these bitches were between 28 and 90 days. Five of the six bitches were reported to show fear and/or anxiety-related behaviours in the months following neutering (Table 3).

**Table 3:** Descriptive data for six bitches that had health codes for pseudopregnancy after neutering surgeries.

Bitch number	Days between estimated ovulation and neutering	Days between neutering and ‘false pregnancy’ code	Clinical signs noted	Treatment provided	Duration of clinical signs	Behavioural signs noted in the bitch’s records
1	39	9	Milk production	Cabergoline from 9 days post-neuter	11 days	Some anxious behaviours first reported four months post-neuter
2	28	5	Inappetent, mammary development, ‘clingy’, nesting	Cabergoline from 8 days post-neuter	Unknown, resolved with one five day course Cabergoline and in <2 months	Anxious when out on walks
3	90	3	Inappetent, nesting, milk production	Cabergoline from 3 days post-neuter	<25 days	Fear towards unfamiliar objects and loud sounds, not observed following home change
4	80	3	Mammary development, milk production, hoarding toys, inappetant	Cabergoline from 2 weeks post-neutering	18 days	None
5	70	10	Nesting, whining, milk production	None	25 days	Some anxious behaviours and body sensitivity noted
6	86	9	Milk production. Swollen discharging mammary continuously until removed four months post-surgery	Amoxicillin Meloxicam Cabergoline Surgical removal of gland	Four months	Some anxious behaviours noted

#### 3.3.2 Behaviours that may indicate pseudopregnancy

##### 3.3.2.1 Behaviour questionnaire trait scores

**Figure 2:**
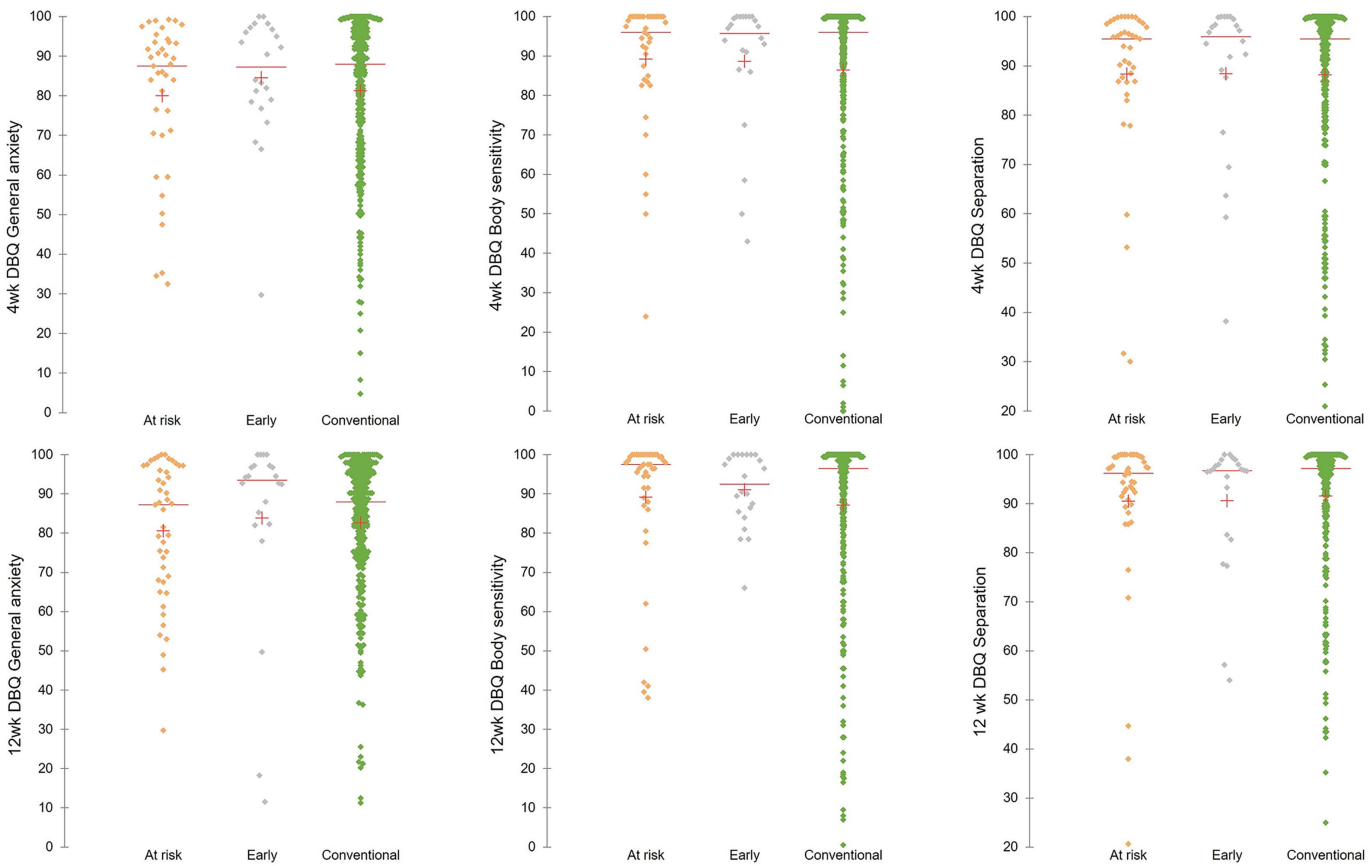
The distributions of 4-week (top row) and 12-week (bottom row) DBQ trait scores for general anxiety (left), body sensitivity (middle), and separation-related behaviour (right) for bitches neutered in the ‘early’, ‘conventional’, or ‘at greatest risk’ (at risk) neutering periods. The mean (red +) and the median (red line) are displayed.

Forty-eight bitches had 12-month PBQ data, 376 bitches had 4-week DBQ data, and 381 bitches had 12-week DBQ data for questionnaires completed at least two weeks post-neutering (Table 4). Data analysis for 12 m PBQ data was not possible due to small numbers of EP (*n* = 0) and GRP bitches (*n* = 5) with data available. Visualisations of trait scores from four- and 12-week DBQ for EP, CP and GRP bitches did not reveal any obvious associations (Figure 2).

**Table 4:** The number of bitches with data available from 12-month PBQs and four- and 12-week DBQs.

Number of bitches	12-month PBQ	Four-week DBQ	12-week DBQ
Number of bitches with data	48	376	381
Number of bitches neutered in the ‘early’ period	0	22	22
Number of bitches neutered in the ‘conventional’ period	43	315	314
Number of bitches neutered in the ‘at greatest risk’ period	5	39	45

None of the models for 4-week (General Anxiety D. F. = 9, Chi-square = 8.031, *p* = 0.531; Body Sensitivity D. F. = 9, Chi-square = 16.323, *p* = 0.060; Separation D. F. = 9, Chi-square = 8.519, *p* = 0.483) and 12-week DBQ trait scores (General Anxiety D. F. = 9, Chi-square = 8.927, *p* = 0.444; Body Sensitivity D. F. = 9, Chi-square = 9.435, *p* = 0.398; Separation D. F. = 9, Chi-square = 5.283, *p* = 0.809) identified significant associations between any factors and behavioural trait scores.

##### 3.3.2.2 Behaviour incidents and dog development plans

Two hundred and seventy-eight bitches (10.9%) had at least one behaviour incident report related to fear or anxiety, and/or aggressive behaviours in the period up to 6 months post-neutering. Thirteen of the 319 EP bitches (4.1%) had at least one behaviour incident in the period up to 6 months post-neutering compared to 220 of the 1,993 CP bitches (11.0%) and 45 of the 231 GRP bitches (19.5%). Bitches had between one and 21 individual behaviour incident reports. The median number of reports for EP and GRP bitches was 3.0 and was 2.5 for CP bitches. There was no significant difference between EP, CP or GRP bitches in the number that had one or more than one behaviour incident report (Chi-square = 2.646, D. F. = 2, *p* = 0.266). Behaviour incidents were reported to first occur from seven to 183 days from date of neutering surgery. There was no significant difference in the day following neutering surgery that the first behaviour incident was reported between groups (*K* = 0.046, *p* = 0.977).

One hundred and thirty-six bitches (5.3%) had at least one dog development plan related to fear or anxiety, and/or aggressive behaviours in the period up to 6 months after neutering. Five of the 319 EP bitches (1.6%) had at least one dog development plan in the period up to 6 months post-neutering compared to 113 of the 1,993 CP bitches (5.7%) and 18 of the 231 GRP bitches (7.8%). Bitches had between one and four individual dog development plans. The median number of development plans for bitches in all groups was 1.0. Chi-square analysis to examine differences between the number of bitches neutered in each period that had one or more than one dog development plans was not possible due to three of six frequencies <5. Dog development plans were reported to first occur from 11 to 182 days from date of neutering surgery. There was no significant difference in the day following neutering that the first behaviour incident was reported between groups (K = 0.463, *p* = 0.793).

When data for behaviour incidents and development plans were combined, 295 bitches (11.6%) had at least one behaviour incident or dog development plan related to fear or anxiety, and/or aggressive behaviours in the period up to 6 months post-neutering. These bitches were neutered between 24 and 218 days from estimated ovulation date. One hundred and nineteen bitches (4.7%) had both (five EP bitches, 99 CP bitches and 15 GRP bitches). Thirteen of the 319 EP bitches (4.1%) had at least one behaviour incident or development plan compared to 234 (11.7%) of the 1,993 CP bitches and 48 (20.8%) of the 231 GRP bitches. One of the six bitches that had health codes for ‘false pregnancy’ after neutering had a behaviour incident and a development plan related to fear behaviours in the period up to 6 months post-neutering.

**Figure 3:**
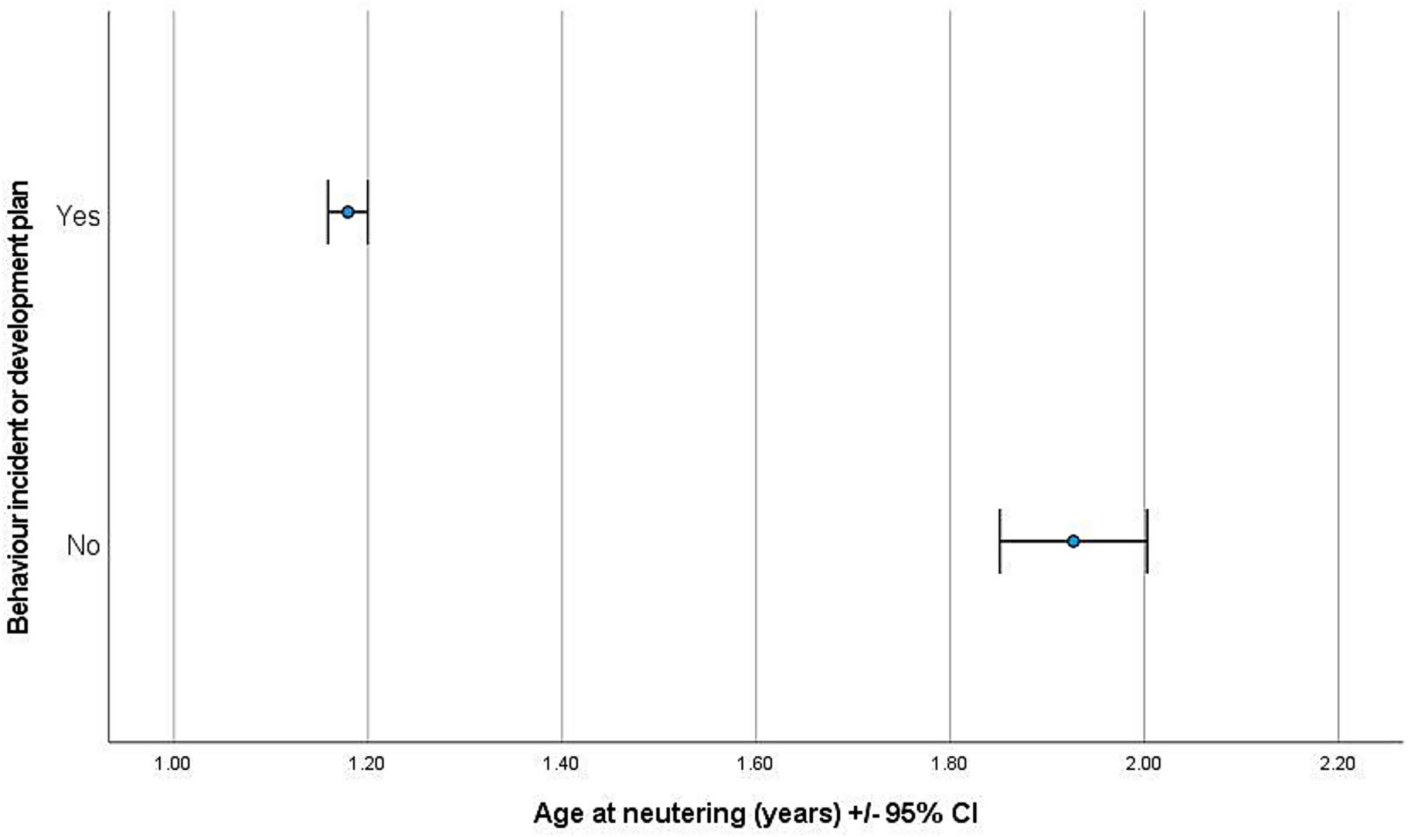
The age at neutering for bitches with and without a behaviour incident and/or development plan for fear/anxiety or aggression-related behaviours in the six months post-neuter.

The logistic regression model was significant (D. F. = 8, Chi-square = 118.995, *p* < 0.001, AUC = 0.660) and all variables were retained. Neutering period (D. F. = 2, Chi-square = 15.092, *p* = 0.001), breed group (D. F. = 5, Chi-square = 23.355, *p* < 0.001) and age at neuter (D. F. = 1, Chi-square = 62.568, *p* < 0.001; Figure 3) were all associated with a bitch having a behaviour incident or development plan. EP bitches (OR = 0.338, 95% CI = 0.175–0.656, *p* = 0.001) and CP bitches (OR = 0.527, 95% CI = 0.371–0.750, *p* < 0.001) had reduced odds of having a behaviour incident and/or development plan related to fear or anxiety, and/or aggressive behaviours than GRP bitches.

For breed group, GR bitches (5.6%) had reduced odds of having a behaviour incident and/or development plan than LR bitches (13.6%, OR = 0.394, 95% CI = 0.222–0.698, *p* = 0.001), GSD and GSD cross bitches (14.9%, OR = 0.438, 95% CI = 0.231–0.832, *p* = 0.012) and ‘Other’ breeds (20.0%, OR = 0.354, 95% CI = 0.143–0.879, *p* = 0.025). First generation cross bitches (10.4%) had reduced odds of having a behaviour incident and/or development plan than GSD and GSD cross bitches (14.9%, OR = 0.600, 95% CI = 0.392–0.920, *p* = 0.019). First (10.4%, OR = 0.539, 95% CI = 0.392–0.742, *p* < 0.001) and second-generation cross bitches (11.5%, OR = 0.648, 95% CI = 0.451–0.932, *p* = 0.019) had reduced odds of having a behaviour incident and/or development plan than LR bitches (13.6%). The equation for the best fit model is provided in the Supplementary material.

### 3.4 Indicators of pseudopregnancy post-neuter for bitches that experienced a pseudopregnancy after the oestrus prior to neutering

Three hundred and thirty-two bitches (13.1%) experienced a pseudopregnancy before neutering; 265 of these (10.4% of total) were pseudopregnancies following the oestrus immediately prior to neutering. Fourteen of these 265 bitches (5.3%) had clinical signs that could be associated with pseudopregnancy mentioned in their health records in the month following neutering. The clinical signs most commonly noted in the health records for these 14 bitches were inappetence (*n* = 6), vomiting (*n* = 4), ‘stressy’/anxious behaviour (*n* = 2). Additionally, being ‘clingy’, ‘destructive’, or showing nesting behaviour and mammary development were each reported once. All 14 were CP bitches neutered between 97 and 242 days from estimated ovulation date (mean = 121.0 ± 9.6 days).

## 4 Discussion

The present study successfully reported comparisons between 319 bitches neutered in the ‘early’ period, 1,993 bitches neutered in the ‘conventional’ period and 241 bitches neutered in the period ‘of greatest risk’. There were no associations between neutering period and the presence of an intraoperative bleed or reports of a pseudopregnancy following neutering in the clinical health records, although clinically coded post-neuter pseudopregnancy was rare, potentially reflecting under-reporting. There were also no associations between neutering period and trait scores for behaviours that may indicate pseudopregnancy on dog behaviour questionnaires. However, there was an association between neutering period and the odds of a bitch having a behaviour incident report and/or dog development plan related to fear or anxiety, and/or aggressive behaviours in the period up to 6 months post-neutering. GRP bitches had higher odds of having a behaviour incident report and/or dog development plan than EP or CP bitches. While the present study was retrospective, and there are some study limitations, the results suggest that there is not an increased likelihood of intraoperative bleeding or indicators of pseudopregnancy post-neutering for large breed bitches neutered in the ‘early’ neutering period compared to those neutered in the ‘conventional’ neutering period.

There are some limitations that should be considered when interpreting results from this study. The study included only large and cross breed bitches from an assistance dog organisation’s population. This may impact the generalisation of findings and clinical applicability for other breeds; further work would be required to confirm these results across bitches of different breeds and from other populations. The study is limited by the unequal number of bitches in each neuter group, with data available for considerably more CP bitches than EP or GRP bitches, along with the uneven distribution in days from estimated ovulation date to neuter for EP bitches, with only five neutered between 0 and 20 days from estimated ovulation. The collection of blood samples to determine ovulation date is not routinely undertaken for all bitches in the organisation, and therefore ovulation date was estimated based on season end date. Season end dates were estimated for some bitches; therefore, ovulation date estimates may be inaccurate by a few days. This may have resulted in some bitches being misclassified into neutering period groups. There was a large number of veterinary practices that performed neutering surgeries for bitches in the study, and the variable was grouped for analysis. It is possible that the veterinary surgeon performing the surgery could influence the likelihood of an intraoperative bleed, however individual surgeon data were not available for inclusion in the study. Future prospective studies would benefit from limiting the number of veterinary surgeons and collecting these data for inclusion as random effects in statistical models. Data for intraoperative bleeds and clinical signs of pseudopregnancy were limited by the data available; due to the retrospective nature of the study, reports of intraoperative bleeding were obtained from clinical records submitted by the veterinarians that performed the surgeries and were reliant on the assessment of the operating surgeon and details being reported. However, veterinarians may be likely to report intraoperative complications, such as bleeding. The lack of a prospective study design also precluded all bitches being examined by a veterinarian for signs of pseudopregnancy post-neuter, which may have resulted in underreporting, however the rigorous health monitoring of the bitches by trained staff and volunteers may have reduced this risk in comparison to retrospective studies in pet dogs or studies reliant on veterinary records alone. In the retrospective study, behavioural data were limited for bitches in the two-week to six-month period post-neutering, and there was a lack of prospectively collected, standardised behavioural data. Behaviour questionnaire data were not available for all bitches, improvements could be made with standardised collection of behavioural data at a specific time post-neutering. The quality of behavioural data may be subject to variability caused by factors not included in this study, such as different observers or participants (23, 24), or by inconsistency in the reporting of behavioural incidents across the organisation. These limitations apply to all neuter period groups, and therefore comparisons between groups remain valid.

Overall, 8.5% of bitches were reported to have an intraoperative bleed when bleeds or oozing from the skin and subcutaneous tissue/fat were included and 5.7% when they were not included. These proportions are higher than reported in a previously published prospective study in bitches of similar breeds (25). However, variation due to veterinary practice in neutering outcomes, reported in the same study, may explain the difference. In the present study, rates of intraoperative bleeding were low for VP1 and VP3, and these practices performed a large number of the neutering surgeries in the previous study (25). In the present study, the majority of surgeries (*n* = 1,891) were performed by ‘Other’ veterinary practices, and the rates of intraoperative bleeds for ‘Other’ veterinary practices were higher (10.5 and 6.8% including and excluding bleeds or oozing from the skin and subcutaneous tissue/fat, respectively). The rates of intraoperative bleeding reported in the present study are similar to other studies; haemorrhage during ovariohysterectomy has been reported in 1.1% (26), 6.4% (16), or between 3.9 and 19.6% of surgeries (17). There was no association between neutering period and intraoperative bleeds, although the proportion of bitches with bleeds was lower for EP than CP bitches. This is perhaps unsurprising as no bitches were neutered in proestrus or oestrus, when the reproductive tract is at its most vascular and risk of bleeding is reportedly higher. Although not significantly different, the highest percentage of bitches with intraoperative bleeds was reported for GRP bitches. The underlying mechanism responsible for this observation has not been elucidated by the authors.

Other authors have suggested that overweight bitches and those with high levels of abdominal fat may be more at risk of difficulties during neutering surgeries (16, 17). However, in the present study, there were no significant differences between bitches that had an ‘ideal’ versus ‘overweight’ BCS in exploring intraoperative bleeding. Significant differences were identified between bitches that had an ‘ideal’ and ‘unknown’ BCS. It is possible that many of the bitches with an ‘unknown’ BCS were overweight, and that influenced the finding. However, the result is limited by the high proportion of bitches (*n* = 1,173, 46.1%) that had an unknown BCS. This is due to the low frequency of BCS being recorded, as well as the method of data collection: for a BCS to be included, it had to be reported within 60 days of neutering surgery.

Very few bitches, six (0.2%) in total, had health codes for ‘false pregnancy’ after neutering. This may reflect an underestimation due to underreporting. The proportion of EP bitches with health codes for ‘false pregnancy’ could perhaps be expected to be higher if there was an increased risk of pseudopregnancy due to neutering during this period, though others have shown that bitches with no history of pseudopregnancy do not necessarily develop overt clinical signs of pseudopregnancy when ovariectomies were performed in the luteal phase (9, 11). However, it is suggested by some authors that pseudopregnancies can manifest as behavioural changes in the months following neutering, with an absence of clinical signs (10, 15). These may not have been as well recognised, or been associated with a pseudopregnancy, and therefore may not have been coded in the clinical records. For this reason, the present study also sought to examine detailed behavioural data for bitches in the months following neutering to identify behaviours that may be indicative of pseudopregnancy.

No differences in scores for behaviour traits related to fear/anxiety were identified between EP, CP or GRP bitches. However, there were associations between neutering period and behaviour incident reports and/or behavioural development plans related to fear or anxiety, and/or aggressive behaviours in the period up to 6 months post-neutering. EP bitches (4.1%) and CP bitches (11.7%) had reduced odds of a behaviour incident report or development plan for these behaviours than GRP bitches (20.8%). Although not significant, it is interesting that the proportion of bitches that had at least one behaviour incident or development plan was lower for EP than CP bitches. While these data were not collected prospectively using a standardised method of behavioural data collection for all bitches, bitches in the organisation that are showing these behaviours are likely to have them reported due to the well-managed training programme and intense scrutiny of individual dogs by the technically trained staff responsible for training the dogs.

These results confirm that neutering during the second part of the luteal phase, when plasma prolactin concentrations are increased, is not to be recommended; likely because removal of the ovaries causes a decrease in progesterone and an increase in prolactin. There is good evidence in several species that prolactin influences behaviour (27–29). For bitches that are neutered when prolactin is elevated, as there are then no ovaries and therefore no progesterone, prolactin may remain elevated and influence behaviour. Some authors have reported that the behaviour that they see in bitches with longer-term behaviours that may be indicative of pseudopregnancy post-neutering can be influenced by administration of the prolactin inhibitor cabergoline (14, 30, 31). Harvey et al. (30) investigated the use of cabergoline for bitches with pseudopregnancy, including eight bitches that were showing behaviours that may be associated with pseudopregnancy, mainly aggression, after neutering. Bitches were reported to have ‘elevated’ prolactin concentrations (mean 5.2 ng/mL, range 2.8–9.1 ng/mL) prior to treatment and were treated with 5 μg (0.1 mL)/kg for 5 days. The results showed ‘good’ clinical responses for six of the eight bitches, along with decreases in prolactin concentrations post-treatment (mean 0.7 ng/mL, range 0.5–1.7 ng/mL), five of which were permanent.

In a separate study, Harvey et al. (14) investigated the use of cabergoline in 32 bitches with signs of pseudopregnancy following neutering. Twelve of the 15 bitches that were exhibiting aggressive behaviours showed ‘fair’ (*n* = 6), ‘good’ (*n* = 4) or ‘excellent’ (*n* = 2) responses to cabergoline. The pre-treatment prolactin concentrations were not reported separately for these 15 bitches, but for all 23 bitches with pre-treatment prolactin measurements, 11 were less than 1.0 ng/mL, three were between 1.0 and 2.9 ng/mL and seven were 3.0–5.9 ng/mL. All bitches were reported to have prolactin concentrations that remained, or decreased to, less than 2.0 ng/mL post-treatment. Harvey et al. (14) suggested that, based on the work of Onclin and Verstegen (4) and Okkens et al. (32), prolactin concentrations less than 10 ng/mL were considered basal. Only one bitch had a prolactin measurement over 10 ng/mL in the study. The authors suggest that prolactin is not solely responsible for inducing pseudopregnancy, but perhaps sensitivity of target tissues, and that although there is no definitive method of diagnosing pseudopregnancy in the absence of clinical signs, the fact that cabergoline treatment was successful may confirm the diagnoses (14). However, cabergoline is also a dopamine agonist (33), and in humans, low levels of dopamine are linked to depression and schizophrenia (34). Therefore, in some cases, any change in behaviour following cabergoline administration may be a result of stimulation of dopamine and be unrelated to elevated prolactin, and potentially unrelated to false pregnancy.

Adding further uncertainty to the association between prolactin and pseudopregnancy post-neutering is the fact that there appear to be associations with other factors. There is a generally accepted breed predisposition in some breeds Gobello et al. (35). Afghan Hounds have been shown to have higher basal plasma prolactin concentrations than some breeds, and a high predisposition to pseudopregnancy (9, 32). Other breeds are also anecdotally suggested to be predisposed to pseudopregnancy, such as Dalmatians, Basset Hounds, and Pointers, while others, such as German Shepherds and Beagles, are not (36). Additionally, previous history of pseudopregnancy has been suggested to impact the likelihood of pseudopregnancy post-neuter. Gobello et al. (11) neutered 11 bitches between 17 and 32 days after the start of diestrus, four of which had a history of previous pseudopregnancy. All four bitches showed clinical signs of pseudopregnancy one week post-neutering alongside significantly increased prolactin concentrations. The same was not shown for the seven remaining bitches with no previous history of pseudopregnancy. In the present study, there were 40 EP bitches, 276 CP bitches and 16 GRP bitches that had a history of pseudopregnancy before neuter, and two of these (one EP and one GRP) had a clinical pseudopregnancy noted in their records after neutering. The low levels observed in the present study may be related to a potential reduced risk in the breeds studied.

Not all bitches respond to elevated prolactin concentrations with signs of pseudopregnancy (36). In a study of six Beagle bitches with no previous history of pseudopregnancy that underwent ovariectomy between 25 and 40 days after ovulation, prolactin concentrations were elevated but only mild signs of covert pseudopregnancy were observed (9). Gobello et al. (11) also reported that bitches with no history of pseudopregnancy did not develop overt pseudopregnancy after neutering during the luteal phase. It is therefore possible that the development of a pseudopregnancy post-neutering is influenced by multiple factors, not only elevated prolactin. Breed, previous history, individual changes in prolactin leading to very large increases in some bitches, differences in susceptibility of the target tissues and the bioactivity and immunoreactivity ratios may all play a role in the development of pseudopregnancy (9). Based on the literature available, it may be recommended to avoid neutering in the early luteal phase for bitches with a history of pseudopregnancy, although for bitches in the present study, there did not appear to be an association.

Further work involving a prospective cohort study to recruit equal numbers of bitches to be neutered in the neutering period groups, using blood samples to determine ovulation date to ensure correct neuter period classification, and to collect serial prolactin measurements and standardised data post-neuter, would offer improvements in methodology in comparison to the present retrospective study design. Future studies, particularly including other breeds, and those that may be predisposed to pseudopregnancy, would be useful to further investigate associations between neuter period and pseudopregnancy. In addition, studies to identify associations between hormonal, anaesthetic, surgical and social factors and the development of pseudopregnancy in neutered female dogs would be beneficial. However, based on the data available, the results suggest no associations between neutering in the ‘early’ neutering period and indicators of pseudopregnancy. Neutering in mid to late-dioestrus is not recommended.

## 5 Conclusion

This study examined data for bitches that were surgically neutered during three time periods following their previous oestrus. Comparisons between bitches neutered in the ‘early’ or ‘conventional’ neutering periods showed no significant differences in the presence of an intraoperative bleed, reports of a pseudopregnancy following neutering, or in behaviours that may indicate pseudopregnancy in the period up to 6 months post-neutering. However, bitches neutered in the ‘at greatest risk’ period had increased odds of reports of behaviours related to fear or anxiety, and/or aggressive behaviours in the period up to 6 months post-neuter. The results suggest that veterinarians in practice, particularly where surgical neutering is required for welfare reasons, such as before rehoming for bitches in shelters, could consider neutering during the ‘early’ period for some breeds of bitches where details of the previous oestrus are known, taking into account breed predisposition for, and previous history of, pseudopregnancy.

## Data Availability

Guide Dogs encourages high quality research to improve the health, temperament and welfare of its dogs. Given the unique nature of this population and the data we collect, Guide Dogs is keen to ensure the integrity of any associated research and prevent misrepresentation. As such, we do not routinely release raw data but will allow its use within high quality research proposals that have been approved under Guide Dogs research governance process. Requests to access the datasets should be directed to Rachel Moxon, canine.research@guidedogs.org.uk.
