# Supplementary material for: Retrospective study examining complications and iatrogenic pseudopregnancy in bitches neutered in different stages of the oestrous cycle: identification of an ‘early neutering window’ in bitches
Source: Front Vet Sci. 2026 Feb 3;13:1774042. doi: 10.3389/fvets.2026.1774042 (PMC12909199; doi:10.3389/fvets.2026.1774042)
Supplement: Supplementary file 1 [file Data_Sheet_1.docx]

Supplementary Material

# Supplementary figure


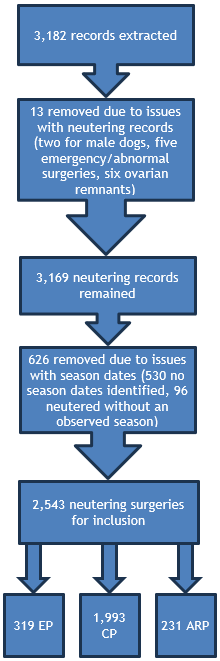


**Supplementary Figure 1.** A flow diagram showing the numbering of neutering records extracted, records that were excluded, the total number of eligible neutering surgeries, the neutering surgeries excluded due to missing season information and the final neutering surgeries included in the study by study group based on neutering period (EP = early period, CP = conventional period, ARP = at greatest risk period).

# The equation for the best fit model for intraoperative bleeds including bleeding or oozing from the skin and subcutaneous tissue/fat

$$Pred(Intraop bleed INCL skin etc) = 1 / (1 + exp(-(-2.439-0.202*Age at neuter+0.694*Vet-VP2-0.383*Vet-VP3+0.800*Vet -Other-1.026*Vet -VP1-0.356*BCS-Overweight-0.456*BCS-Unknown)))$$

# The equation for the best fit model for intraoperative bleeds excluding bleeding or oozing from the skin and subcutaneous tissue/fat

$$Pred(Intraop bleed EXCL skin etc) = 1 / (1 + exp(-(-2.333-0.278*Age at neuter+0.086*Breed-First gen GRxL or LxGR+0.431*Breed-GSD and crosses+0.325*Breed-GR+0.543*Breed-LR+0.302*Breed-Other-0.401*Vet-VP4+0.305*Vet-VP3-0.997*Vet-VP3-1.291*Vet-VP1-0.207*BCS-Overweight-0.381*BCS-Unknown)))$$

# The equation for the best fit model for a bitch having a behaviour incident or development plan

$$Pred(Behav incident OR plan for fear/aggression (1,0) within 6m post-neuter) = 1 / (1 + exp(-(-0.744-0.692*Age at neuter+0.184*Breed Grouped-2ndGenBackX+0.510*Breed Grouped-GSDandXs-0.314*Breed Grouped-GR+0.617*Breed Grouped-LR+0.724*Breed Grouped-Other-1.083*Neuter period-Early-0.640*Neuter period-Normal)))$$
